# The Evolution of Transjugular Intrahepatic Portosystemic Shunt: Tips

**DOI:** 10.1155/2014/762096

**Published:** 2014-03-18

**Authors:** Fabrizio Fanelli

**Affiliations:** Vascular and Interventional Radiology Unit, “Sapienza” University of Rome, Viale Regina Elena 324, 00161-Rome, Italy

## Abstract

Since Richter's description in the literature in 1989 of the first procedure on human patients, transjugular intrahepatic portosystemic shunt (TIPS) has been worldwide considered as a noninvasive technique to manage portal hypertension complications. TIPS succeeds in lowering the hepatic sinusoidal pressure and in increasing the circulatory flow, thus reducing sodium retention, ascites recurrence, and variceal bleeding. Required several revisions of the shunt TIPS can be performed in case of different conditions such as hepatorenal syndrome, hepatichydrothorax, portal vein thrombosis, and Budd-Chiari syndrome. Most of the previous studies on TIPS procedure were based on the use of bare stents and most patients chose TIPS 2-3 years after traditional treatment, thus making TIPS appear to be not superior to endoscopy in survival rates. Bare stents were associated with higher incidence of shunt failure and consequently patients required several revisions during the follow-up. With the introduction of a dedicated e-PTFE covered stent-graft, these problems were completely solved, No more reinterventions are required with a tremendous improvement of patient's quality of life. One of the main drawbacks of the use of e-PTFE covered stent-graft is higher incidence of hepatic encephalopathy. In those cases refractory to the conventional medical therapy, a shunt reduction must be performed.

## 1. Introduction

Portal hypertension is the result of pressure increase within the portal vein when the blood flowing through the liver is blocked. Increase of pressure usually leads to the development not only of varices in the esophagus and stomach but also of ascites [1].

The most common cause of portal hypertension is cirrhosis or liver scarring [2]. Cirrhosis results from the healing of a liver injury provoked by hepatitis, by alcohol abuse, or by any serious liver damage. In cirrhosis, blood flowing through the liver is obstructed by the scarred tissue that slows down its forward movement [3, 4].

Thrombosis and clotting in the portal vein are equally responsible for portal hypertension.

Portal hypertension can also be related to a prehepatic disease, such as inflammation of the umbilical vein in early infancy, resulting in portal vein thrombosis and cavernomatous transformation. A block in the portal flow located before the sinusoids of the liver does not create an increased portal hypertension and usually causes neither a disturbance in the function of hepatocytes nor ascites [2, 3].

There also exists a form of portal hypertension caused by blockage of effluent blood from the liver (Budd-Chiari syndrome and venoocclusive disease) that is related to ischemic changes in the hepatocytes and is accompanied more often by ascites than variceal bleeding [2–4].

Patient with portal hypertension and liver cirrhosis usually reports a chronic affection of the liver by hepatitis B and hepatitis C or alcohol abuse. On physical examination, unless the patient presents with bleeding from esophageal varices or with ascites, the diagnosis of portal hypertension may be suspected only from indirect signs. Multiple spider nevi on the skin usually indicate portal hypertension as do dilated veins on the anterior abdomen. A hard liver on palpation and an enlarged spleen have to lead us toward a possible diagnosis of portal hypertension. An enlarged spleen is also frequent at ultrasound examination [1, 3].

Since Richter's description in the literature in 1989 [5] of the first procedure on human patients, transjugular intrahepatic portosystemic shunt (TIPS) has been worldwide considered as a noninvasive technique to manage portal hypertension complications [6–8]. By diverting blood flow from the portal venous system to the systemic circulation, TIPS succeeds in lowering the hepatic sinusoidal pressure and in increasing the circulatory flow, thus reducing sodium retention, ascites recurrence, and variceal bleeding [9, 10].

Refractory ascites is associated with a severe prognosis in patients with liver cirrhosis, firstly, because 1-year survival is less than 50% and risk of complications (such as spontaneous bacterial peritonitis, hepatorenal syndrome, and dilutional hyponatremia) is very high. Moreover, these patients paradoxically have low Model for End-Stage Liver Disease (MELD) scores despite their high mortality rate and consequently they hold a low position on national transplant listings [11].

Recent studies have revealed a reduced mortality in patients undergoing TIPS placement, compared with those receiving serial large-volume paracentesis procedures, with one-year survival rates ranging from 63% to 80% [12–14].

Large-volume paracentesis is safe and easily performed with the advantage of immediate relief from pain but it has a negative effect on systemic hemodynamics and renal function and this often limits its use as a long-term treatment [15, 16].

Several prospective randomized controlled trials were conducted in the last years to compare repeated paracentesis with TIPS insertion. Results showed a significant advantage over ascites control and a longer survival after performing TIPS [17–19].

Bleeding due to portal hypertension is one of the most important complications of disease of the liver and its related vessels. It often occurs without precipitating factors and usually presents as massive and painless hematemesis. It rarely occurs as chronic bleeding causing anemia. Another possible manifestation is enterorrhagia. The most common and most life-threatening emergency is variceal bleeding which has significant mortality and high risk of rebleeding [20–22].

Esophagogastric varices are a very severe condition for the high and often fatal risk of bleeding.

The prevalence of gastroesophageal varices in cirrhosis varies between 30% (patients with compensated cirrhosis) and 70% (patients with decompensated cirrhosis) [21].

The risk of bleeding from gastroesophageal varices is approximately 30% within the first year of their identification. Twenty percent of cirrhotic patients with acute variceal hemorrhage die within 6 weeks. The rebleeding rates range from 30% to 40% at 6 weeks and the mortality from rebleeding reaches 30%. Optimal management of variceal hemorrhage requires a multidisciplinary approach, involving a team of gastroenterologists, hepatologists, hematologists, critical care physicians, surgeons, and interventional radiologists. The principal components of therapy include airway maintenance, hemodynamic stabilization, control of the variceal hemorrhage, and alteration of the hemodynamic effects of portal hypertension [20–22].

Treatment options for ongoing or past bleeding due to portal hypertension can be divided according to the basic mechanism of action. One strategy is targeted at the actual bleeding source and interventions are performed mainly by endoscopy or surgery. The second strategy concentrates upon the reduction of portal pressure and currently is represented by surgical or radiological shunts.

Endoscopic variceal treatment remains the predominant method of preventing and treating recurrent gastrointestinal bleeding. But endoscopic therapy cannot fundamentally resolve the problem of portal hypertension, and as patients are often unable to tolerate repeated therapies, a high rate of rebleeding is reported [20, 21].

Endoscopy is performed early in the course of management, in an attempt to localize the bleeding site and treat the varices. Generally, the following 2 types of endoscopic-guided interventions are used for controlling variceal hemorrhage: endoscopic variceal banding and variceal sclerotherapy. Neither is more effective in controlling bleeding; many endoscopists, however, favor variceal banding, as the banding devices allow for rapid placement. Nonetheless, endoscopy has limitations that include failure to localize all bleeding sites because of extensive ongoing hemorrhage, inability to treat all bleeding sites, and failure of banding to control hemorrhage. The hemodynamic effects of portal hypertension may be modified through the use of certain systemic drugs. The intravenous infusion of vasopressin/terlipressin decreases mesenteric arterial flow and thereby decreases the portal venous inflow. Selective *β*-blockers, such as propranolol and nadolol, are used to decrease portal hypertension. Placement of a Sengstaken- Blakemore tube is designed to temporarily control variceal hemorrhage with tamponade; this is accomplished by the inflation of the 2 balloon components of the tube, one within the stomach and the other in the esophagus. Generally, it is used only in emergencies where variceal hemorrhage is impossible to control by other means, as ulceration and rupture of the esophagus and/or stomach are recognized complications. These various methods, either alone or in combination, are effective in controlling acute variceal hemorrhage in 80%–90% of patients [20–23].

Patients who do not respond to these measures are referred for flow-diversion (or rescue) therapies, which include transjugular intrahepatic portosystemic shunt (TIPS) and surgical portosystemic shunts with or without splenectomy [21].

TIPS procedure, that reduces portal pressure, is the best choice to prevent and control esophageal and gastric variceal bleeding [22].

Two randomized studies and one retrospective surveillance study compared TIPS with medical treatment for acute bleeding [23–25].

Monescillo compared high-risk patients with a pressure gradient above 20 mm Hg measured within 24 hours who received TIPS or medical treatment. TIPS group had a better outcome. Compared with the medical group, the TIPS group had a significantly better outcome with respect to treatment failure, transfusions, need for intensive care, and in-hospital and 1-year mortality [24].

In the second study by García-Pagán [25] high-risk patients with Child-Pugh class B and acute variceal bleeding at index endoscopy or class C were randomized within 72 h after admission to receive TIPS or medical treatment using *β*-blocker plus nitrate or endoscopic band ligation if irresponsive to drugs. The early TIPS group had a significantly lower rebleeding rate (3% versus 45%) and a better survival (1-year: 87.5% versus 61.3%). In addition, a recent economic modelling of early TIPS in high-risk patients with acute variceal bleeding reported cost effectiveness of TIPS procedure compared with standard therapy, and on the basis of the final results the Baveno V conference in 2010 recommended considering early TIPS (within 72 h) in patients with high risk of treatment failure [26].

Bleeding from gastric varices may sometimes occur even with a low portal pressure gradient [27]. In these patients, the rationale for a decompression alone cannot be given and TIPS alone without embolization cannot be considered the optimal solution. This is confirmed by the finding that TIPS improves mortality only in patients with pre-TIPS pressure gradients above 12 mm Hg [28]. Nevertheless, TIPS was superior to endoscopic embolization as demonstrated in controlled study [29–31].

It has been confirmed that, with respect to prevention of recurrent bleeding, TIPS is better than medication therapy. The postoperative rebleeding rate was 12%–22% in TIPS, and it is even lower with the use of coated stent. For endoscopic therapy, however, the rebleeding rate is much higher (20%–43%). Recently, a study by García-Pagán et al. [25], comparing the use of Viatorr stent-graft TIPS with drug combined endoscopic variceal ligation treatment, showed that in the 16 months of follow-up, only one case of recurrent bleeding occurred in the TIPS group as opposed to 14 cases in the other groups (3.1%* versus *45.2%, *P* = 0.001). In this study, 17.2% of patients in the TIPS group had recurrent bleeding. Cases of rebleeding caused by stenosis of the stent were mostly seen in patients with bare stents. In the endoscopy group, 50% of the patients had recurrent bleeding. This further confirmed that TIPS is superior to endoscopic therapy in prevention of recurrent bleeding and coated stents can further reduce the incidence of recurrent bleeding by lowering the rate of stent stenosis.

A large number of clinical studies and meta-analyses indicate that TIPS procedure is not superior to endoscopic therapy with respect to improvement of survival time. This is the main reason why TIPS is used as a rescue option after the failure of the traditional therapeutic method. However, although rescue TIPS procedure can effectively control acute bleeding, the postoperative one-year survival rate is only 27%–55% [20–26].

Most of the previous studies on TIPS procedure were based on the use of bare stents and most patients chose TIPS 2-3 years after traditional treatment, thus making TIPS appear to be not superior to endoscopy in survival rates. In the study by García-Pagán et al. [25], the patients in the TIPS group received TIPS with coated stent at the first incidence of bleeding during the early stages. Results showed that early and middle stage survival rates were much higher in TIPS group than in drug combined endoscopic group.

TIPS can be performed also in case of different conditions such as hepatorenal syndrome, hepatic hydrothorax, portal vein thrombosis, and Budd-Chiari syndrome [32–36].

Hepatic hydrothorax occurs in patients with ascites when there is a direct communication between peritoneal and pleural cavities. In most patients, the defect is in the diaphragm that overlies the dome of the liver. Hepatic hydrothorax is the consequence of an accumulation of ascitic fluid that migrates through the diaphragmatic defect [32, 33, 37].

Budd-Chiari is characterized by the absence of hepatic veins and can be associated with portal vein thrombosis, thrombosis of inferior caval vein, and renal failure [34, 38].

The obstruction can occur anywhere from the small hepatic veins to the right atrium of the heart. This causes the dominant clinical features of abdominal pain, hepato- and splenomegaly, ascites, and oesophageal varices and the development of fulminant hepatic failure.

The best way to improve the hepatic blood flow and function is to create a shunt, that is to say, an artificial outflow via the portal vein bed. The shunt has the great advantage of reducing portal hypertension and relieving from splanchnic congestion [35].

Budd-Chiari patients require a transcaval TIPS, with direct puncture of the liver parenchyma. A longer shunt is created that generally needs more than one stent implantation. As a high risk of complications is reported, such as perforation of the liver capsule without or with intraperitoneal hemorrhage in 33 and 1%–2% of the procedures, respectively, ultrasound guidance is considered mandatory [36, 38–40].

Besides the technical challenge correlated with TIPS in Budd-Chiari syndrome, mortality rate is very promising with 1-year survival rates between 71% and 93% and 5-year rates between 74% and 88% [36, 39].

Up to 28% of patients with cirrhosis suffer from portal vein thrombosis [41]. A hypercoagulative state is an exception indicating that hemodynamic factors play the primary role. Warfarin treatment results in complete resolution of the thrombus in 39% of cases, partial resolution in 43%, and no change in 18% [42].

Luca et al. [41] described the effect of TIPS on portal vein thrombosis in cirrhosis: 87% of patients improved with a complete recanalization. Long-term outcome was excellent with 24-month survival of 81%.

TIPS treatment is valid also in patients with cirrhotic or noncirrhotic portal vein thrombosis with cavernomatous transformation [43–47].

TIPS placement may be technically difficult in patients with a cavernous transformation of the portal vein and, some years ago, the procedure was considered contraindicated [46]. More recently some authors presented their first satisfactory results by showing that TIPS is possible in patients with portal cavernoma although a lower feasibility must be expected.

Today the number of reports available is still limited to small groups of patients, to assorted cases with and without cirrhosis, and to patients with portal vein thrombosis of neoplastic origin [44–47]. Performing TIPS in cavernous portal vein occlusion is undoubtedly challenging.

Transjugular access to the portal system may cause the puncture of a cavernous collateral rather than of a normal intrahepatic branch. This event limits not only the ability to navigate but also the shunt patency as a consequence of a very small flow and of an inadequate portal decompression [46].

Fanelli et al. [43] reported their experience in 13 patients with portal cavernoma. Eight patients were candidates for TIPS placement because of bleeding related to portal hypertension that conventional treatment could not control. Two patients had symptoms of intestinal ischaemia for acute superior mesenteric vein thrombosis despite oral anticoagulation. In one patient with concomitant Budd-Chiari syndrome, TIPS was indicated for ascites refractory to diuretic therapy. In the other two patients, TIPS was planned because a lifelong oral anticoagulation therapy was necessary as large oesophageal varices at high risk of bleeding were present and associated with gastric varices. TIPS was successfully implanted in 10 patients (83.3%) with a significant reduction of the portosystemic pressure gradient. In 2/12 patients, TIPS placement failed because catheterization of the extrahepatic portion of the thrombosed/sclerotic portal vein was not achieved. No patient died periprocedurally or within 30 days from the procedure. According to the author porta cavernomatosis can be no longer considered a contraindication to TIPS.

Another technique, described by Jourabchi et al. [48], is to combine a percutaneous portal access with a transjugular one. Percutaneous transhepatic right portal vein access allows performing easily recanalization of the occluded portal vein and has the advantage of a secure portal access and of a direct angle of approach to the occlusion. In addition, it allows using different combinations of wires and catheters to smoothly cross the occluded segment. Once the portal vein is recanalized via the transhepatic approach, the final steps are portal connection and inflation of a balloon catheter within the right portal vein access. From the traditional jugular approach, the needle is advanced from the hepatic vein to the portal system using the dilated balloon as a target point. The balloon rupture confirms the puncture of the portal vein.

Summarizing indications are recurrent variceal hemorrhage in patients who have failed endoscopic and medical therapy, refractory ascites, Budd-Chiari syndrome or hepatic venoocclusive disease, and hepatic hydrothorax.

Other less common indications include the poorly understood entities of portal (congestive) gastropathy and the hepatorenal syndrome. TIPS may also be performed as a bridge to liver transplantation in the cirrhotic patient.

Generally speaking, absolute contraindications to TIPS include right heart failure and pulmonary arterial hypertension. TIPS is of unclear survival benefit in patients with severe liver failure (Child-Pugh class C cirrhosis, Model for End-Stage Liver Disease score >22, serum bilirubin >3 mg/dL). Other relative contraindications include hepatic encephalopathy (which may worsen following TIPS creation), polycystic liver disease (technically challenging with a high incidence of hemorrhagic complications), active sepsis (poor outcomes), and chronic organized portal vein thrombosis (technically challenging for successful TIPS creation) [46, 49].

TIPS success is usually classified as technical, hemodynamic, and clinical.

Technical success means correct creation of a shunt between the hepatic vein and the intrahepatic branch of the portal vein.

Hemodynamic success refers to a satisfactory post-TIPS reduction of the portosystemic gradient. A common hemodynamic endpoint, especially when managing bleeding varices, is a portosystemic gradient of 12 mm Hg [50].

Clinical success is correlated with relief from symptoms and long shunt patency.

TIPS procedure was first described by Josef Rösch in 1969 [51] and first performed on a human patient by Dr. Ronald Colapinto in 1982 [52] but it became successfully reproducible only when endovascular stents started developing. The first successful TIPS was realized by M. Rössle, G. M. Richter, G. Nöldge, and J. Palmaz at the University of Freiburg [53, 54].

Ever since an incredible improvement of this technique has been observed especially during the last 10 years, the technique itself is still more or less as it was in the past but many changes have been made to the characteristics of the stent to achieve a correct implantation and to keep the shunt open [55–60].

The blind PV puncture is a critical moment during TIPS procedure because of the high potential risk of procedural complications involving patient's morbidity.

To overcome these possible complications, different techniques have been studied to correctly visualize the portal venous system such as direct transhepatic catheterization of the portal vein, superior mesenteric artery (SMA) angiography, real-time sonographic guidance, placement of a metallic marker, and refluxing contrast medium into the portal vein with wedged hepatic venography [47].

Although these techniques help improve the guesswork of puncturing the portal vein, they increase the length of the procedure and are often associated with further complications.

Wedged hepatic venography is performed to delineate the angiographic relationship between the selected hepatic vein and the portal venous system as well as to provide a target for transhepatic needle puncture during TIPS insertion [58]. The order in which the portal branch vessels opacify with contrast material during wedged hepatic venography can help confirm the identity of the catheterized hepatic vein if uncertainty exists (e.g., right hepatic vein versus middle hepatic vein). After a right side hepatic venous wedged injection, the right portal vein is expected to opacify first, with subsequent filling of the left portal vein and the main portal vein. Instead, after a middle hepatic venous wedged injection, the left and right portal veins are expected to fill simultaneously, with later opacification of the main portal vein.

Wedged hepatic venography may be performed via a catheter or sheath directly wedged against the liver parenchyma or via a balloon occlusion catheter. Direct wedging of a catheter or sheath against the hepatic parenchyma has the advantage of being relatively quick and easy to perform but it has the great risk of direct liver injury for the proximity of the injection to the liver parenchyma and for the direct pressure of the contrast injection on it. Wedged venography, performed via a balloon occlusion catheter, dissipates the pressure of the injected contrast material over a large surface area of the liver parenchyma and reduces the risk of liver injury. However, a more proximal injection from a balloon occlusion catheter may result in suboptimal portal venous opacification if contrast outflow is present via intrahepatic venovenous collateral channels [46, 49].

The use of dilute iodinated contrast material for wedged hepatic venography guarantees excellent contrast resolution and high visibility of portal venous structures.

Carbon dioxide has a very low viscosity and easily results in retrograde sinusoidal diffusion and portal venous opacification, with lower risk of liver injury. This agent, almost inexpensive, gives no contrast reactions or anaphylaxis and has no renal toxicity. The injected volume of carbon dioxide usually ranges from 30 to 40 mL, depending on the degree of portal venous opacification achieved during the injection. Approximately 2 minutes are allowed to elapse between carbon dioxide injections to ensure an adequate respiratory clearance [46].

A direct intrahepatic portocaval shunt technique (DIPS) was introduced in 2001. It is based on an intravascular ultrasound-guided puncture directly from the inferior vena cava (IVC) to the portal vein via the caudate lobe of the liver. The main advantages of DIPS procedure are direct visualization of the needle track during portal vein puncture, thus eliminating the blind portal vein puncture of the TIPS technique, and significant improvement of procedural safety and effectiveness [47, 61, 62].

In addition, DIPS removes the most common cause of TIPS failure, namely, hepatic vein stenosis, because the shunt extends directly from the portal vein to the inferior vena cava, avoiding the hepatic vein outflow tract.

Hoppe et al. [47] reported their experience performing DIPS procedure on 19 patients. Technical success was achieved in all patients and a marked reduction of the pressure gradient was described from 23.2 to 8.2 mm Hg (mean values). However, a 6% rate of intraperitoneal bleeding was reported because one patient had a segment of the stent-graft incorrectly deployed in the extrahepatic tract.

TIPS procedure is generally performed via the right internal jugular vein that usually provides easy and straight access to the inferior vena cava. This approach is preferable because there is no lymphatic duct in most of the cases and the apex of the right lung is lower that the contralateral. Besides the reported success ranging from 75% to 99%, sometimes this approach is not possible and consequently TIPS must be performed via another access [60]. Left internal jugular vein can also be used but we have to keep in mind that the course from the left side through the left brachiocephalic vein to the superior vena cava is angled. This may cause thoracic pain from stretching of the mediastinal vessels with stiff steel cannula. In some cases also the external jugular vein can be used. Femoral venous approach technique, accurately described and often discussed, provides a good access site in case of occluded jugular approach [63, 64].

But it is stent patency that represents the big challenge of this technique. High rate of shunt obstruction, due to intima hyperplasia (50%–60% at one year and 70%–85% at two years), requires a constant surveillance and frequent expensive revisions [65–67].

Many patients, in fact, during the follow-up period, usually need more than one revision, sometimes also 3 or 4, and this not only increases procedural risks but also reduces patient's quality of life. Just for this reason the number of procedures dramatically decreased in the late 1990s when many physicians preferred to resolve portal hypertension with medical therapy or surgery.

Transjugular intrahepatic portosystemic shunt dysfunction has been attributed to three different mechanisms: acute thrombosis within the stent; pseudointimal hyperplasia secondary to the biliary leaks of the lacerated bile ducts into the shunt lumen; and intimal hyperplasia in the outflow hepatic vein.

This problem was overcome when several experimental and clinical studies concentrated their research on improving covered stent-grafts whose use significantly bettered long-term patency of TIPS shunt.

Different materials [68–71] were analyzed and proven to have bare stents so adequately covered to increase patency rate. In the end several experimental studies with animals highlighted that the best results had been achieved with stents covered with polytetrafluorethylene (PTFE) as reported by Nishimine [72] and confirmed by Saxon [73], Haskal [70, 74], and Andrews [75].

More recently, the routinely use of extended-polytetrafluoroethylene (e-PTFE) covered stent-grafts has reduced intimal hyperplasia and remarkably prolonged shunt patency if compared to shunt patency in patients treated with bare metal stents [76–79].

At the beginning of the covered stent era some difficulties were obviously observed, for most part secondary to an inadequate learning curve [80].

Nashimine et al. [72], who conducted experimental studies on animals, were the first to stress the importance of completely covering the intrahepatic tract and their statement brought a “revolution” to the TIPS world because, for instance, among “Tips and Tricks,” for the best use of a bare stent there was the advice to choose a short stent especially when patients were candidates to liver transplant (OLT).

An open debate has been kept existing about the calibre selection of the Viatorr (WL Gore & Associates, Flagstaff, AZ, USA) stent-graft. Being overall familiar with bare stents, many operators initially preferred to use a 12 mm Viatorr but they soon became aware of a high incidence of HE correlated with the complete absence of intimal hyperplasia that in bare stents was responsible for the progressive narrowing of the stent inner lumen [80, 81]. Thus, 8 and 10 mm stent-grafts became the most commonly employed. However, a randomized trial, conducted by Riggio et al., [82] reported that the best outcomes had been achieved using 10 mm devices. The trial was preterm concluded because the pressure gradient was not sufficiently reduced by 8 mm TIPS.

TIPS creation can be associated with a high rate of hepatic encephalopathy (HE) ranging from 3 to 35%, especially in case of a marked reduction of the portosystemic gradient (PSG < 12 mm Hg) and to overcome this problem several studies have been conducted [83–85].

Incidence and severity of hepatic encephalopathy are higher during the first month but they progressively decrease since the shunt tends to spontaneously reduce its diameter. This is confirmed by the increase of PSE index and ammonia levels during the follow-up of those patients who have undergone a TIPS revision for shunt stenosis [86].

Hepatic encephalopathy is a complex neuropsychiatric syndrome that accompanies severe liver disorders. Acute hepatic encephalopathy is usually associated with fulminant hepatic failure and with the rapid development of hepatic come. Patient dies of cerebral edema. Chronic hepatic encephalopathy has its origin in the insufficient detoxifying function of the liver because, due to portal hypertension, nitrogenous products originating in the gut bypass the liver and enter the systemic circulation. Chronic hepatic encephalopathy is clinically less obvious and is often reversible. The most serious cases manifest clinically in the form of confusion, agitation, or other psychiatric disturbances. In advanced stages of hepatic encephalopathy, patient lapses into stupor or coma [83, 85, 86].

In general a dedicated medical therapy with lactulose, nonabsorbable antibiotics, and an appropriate protein restricted diet (1 gr/kg body weight) is able to reduce and control hepatic encephalopathy [86].

However, when patients do not respond to the medical therapy, a reduction/occlusion of the shunt seems to be the best solution for managing this uncomfortable situation [87, 88].

Shunt occlusion performed with different materials such as nonreadsorbable materials or occlusion balloons has been reported by Rose and Katz [89], Kerlan et al. [90], and Haskal et al. [91].

This technique is unfortunately associated with a high risk of variceal rebleeding, consequent to the irreversible increase of portal pressure as well as of portal thrombosis. Kochar reported a shunt occlusion using an inferior vena cava filter with or without coils embolization [92]. But also this technique showed a very high incidence of procedure related complications such as portal and mesenteric vein trombosis with intestinal infarction.

On the basis of the data available, partial occlusion of the TIPS shunt appears to be the most reliable method because it allows reversal of flow-related complications and control of portal hypertension.

Several techniques have been described, using different types of bare and covered stents with or without the adjunction of coils or other materials but each of them has shown at least one unsatisfactory outcome or unexpected complication and all of them have always been performed in a homemade fashion [93, 94].

Haskal and Middlebrook [95] constrained a Wallstent (Boston Scientific, Natick, MA, USA) with a 3-0 silk suture in an hourglass shape with a constrained diameter of 5 mm. The author attributed the blood flow reduction through the stent to the increased friction and turbulence created by the interposed stent mesh.

Madoff et al. [96] described in 2003 the use of constrained stent-grafts (Wallgraft, Boston Scientific, Natick, MA, USA) to treat six patients. A clinical improvement was achieved within 72 hours. But polyethylene terephthalate (PET) stents provoked a thrombogenic and inflammatory response that led to a precocious shunt occlusion.

The use of expandable-polytetrafluoroethylene (e-PTFE) covered stents seems to be very effective, as reported by Quaretti et al. [97] and Cox et al. [98]. Both authors described a case of hepatic encephalopathy after TIPS resolved with reduction of the shunt lumen by using an “hourglass” self-expanding stent-graft.

One of the leading scientific works in the literature was published by Fanelli et al. [94] who reported 12 cases of hepatic encephalopathy refractory to conventional medical therapy and successfully managed by reducing the shunt lumen with a commercially available e-PTFE balloon expandable stent-graft (Jostent, Abbott Vascular) released inside the Viatorr with an “hourglass” shape. The Jostent determines an immediate reduction of the flow within the initial TIPS and a rapid increase of the portosystemic gradient value. Moreover, the calibre of the shunt can be adjusted (increase in diameter only) during the follow-up according to the patient's clinical conditions.

## 2. Variceal Embolization

Embolization of gastroesophageal varices is performed after TIPS insertion embolization may be performed before or after stent insertion. Pre-TIPS embolization has the advantages of improved variceal filling and visualization as well as reduced risk of systemic coil migration and nontarget embolization. Post-TIPS embolization has the advantage of the ability to assess variceal decompression after TIPS placement. Nonfilling varices may not require embolization after shunting.

As TIPS clinical success is strictly associated with shunt patency, a regular follow-up is mandatory for early detection and timely correction of any malfunction [99–102].

Color-Doppler Ultrasound (USCD), portography with pressure measurement, and Multidetector CT (MDCT) can assess shunt patency.

USCD is a safe, noninvasive, inexpensive, easily performed procedure with a sensitivity of 53–100% and a specificity of 62–98% [103–105]. It allows the accurate analysis of intrahepatic flow velocity and direction as well as stent patency evaluation. But USCD is time consuming and mostly dependent on the operator's skill. Moreover, the day after the procedure, a correct evaluation of the stent-graft is not possible for the presence of bubble air within the e-PTFE graft [99].

Carr analyzed USCD use in TIPS performed with e-PTFE covered stent-grafts. A retrospective study on 52 patients showed that venography and USCD were concordant in 8 of 15 paired studies (53%). The conclusion was that routine USCD is not effective for long-term surveillance of e-PTFE covered stent-grafts [106].

Portography, with measurement of portal venous pressure gradient, is traditionally considered the gold standard for the evaluation of shunt patency but it is an invasive and expensive technique unfit for routine follow-up and is to be recommended only in case of shunt dysfunction or when a revision is required.

With the introduction of spiral scanners, MDCT was proposed as a screening modality for TIPS follow-up. In fact, the use of axial and multiplanar images permits a complete evaluation of the shunt and of the intrahepatic flow rate [101, 102].

Chopra [101] described helical CT angiography as an excellent detector of shunt abnormalities with a sensitivity of 97%, a specificity of 89%, and an accuracy of 94%. In case of significant abnormalities, the results were 92% sensitivity, 77% specificity, and 84% accuracy.

Our experience suggests a sensitivity of 95.2% and a specificity of 96.6% for MDCT, higher than data recorded for USCD: sensitivity 90% and specificity 75%. The Positive Predictive Value (PPV) and the Negative Predictive Value (NPV) were, respectively, as follows: 90.9% and 98.2% for MDCT; 54.5% and 95.7% for USCD [102].

TIPS can be correlated with major and minor complications.

Major complications are as follows: hemoperitoneum, stent malpositioning, hemobilia, hepatic infarction, resistant hepatic encephalopathy, and death rate ranging from 3 to 5% [107–110].

Minor complications are as follows: biliary duct puncture, gallbladder puncture, right kidney puncture, transient pulmonary edema, transient hepatic encephalopathy, and transient renal failure. Such complications may occur in 4–8% of the cases [107, 108, 111].

Biliary fistula formation is an infrequent complication of hepatic parenchymal puncture occurring with an incidence of less than 5% [112]. Although puncture of the bile ducts or gallbladder is usually well tolerated, fistulous communication between biliary and vascular systems may result in hemobilia, cholangitis, sepsis, and stent infection. If a fistula develops between the biliary system and stent, marked pseudointimal hyperplasia and secondary stent occlusion may result.

The occurrence of fistulous communication between the biliary or arterial system and portal vein may be decreased by practicing controlled needle passage and reducing the number of needle punctures. Internal (plastic stent) or internal-external (drainage catheter) biliary diversion may be used to address biliary-vascular fistulas and embolotherapy may be used for arteriovenous or arterioportal fistula formation, particularly in cases of hemobilia. Fistulous communication between TIPS stents and the biliary system has been successfully treated by a covered stent relining the hepatic parenchymal tract [112].

Inadvertent puncture of the hepatic artery or its branches during TIPS insertion is uncommon, occurring with an incidence of approximately 6% [112]. In general, transjugular hepatic arterial puncture carries low clinical significance because the rate of symptomatic arterial injuries is less than 2% [60, 112]. Interestingly, a study comparing the incidence and clinical implication of hepatic arterial puncture between TIPS access sets found no significant differences in low arterial puncture rates or frequency of angiographic arterial injury between 16-gauge and 21-gauge transjugular access needles. Potential complications of hepatic arterial puncture include hemorrhage, pseudoaneurysm formation, vascular dissection or occlusion, and arterioportal fistula, which may result in worsening of preexisting portal hypertension. Nontarget organ injury is a rare complication related to the transhepatic needle puncture phase of the TIPS procedure. Nontarget organs that are at risk of injury include the gallbladder, right kidney, duodenum, and colonic hepatic flexure. As the number of needle passes for portal venous access increases, the incidence of nontarget organ injury also increases. The overall rate of nontarget organ injury is low, with the most commonly injured organ being the gallbladder artery.

Hepatic ischemia and infarction occasionally complicate TIPS placement. Infarction is thought to be related to the shunting of flow from the portal vein into the systemic venous circulation, with a reduction in sinusoidal flow [113]. Hepatic perfusion after TIPS depends on the arterial buffer reserve which is negatively correlated with the Child-Pugh score. Stent compression of the hepatic artery also has been shown to cause hepatic ischemia or infarction [114]. Hepatic infarction is a relatively uncommon complication related to TIPS [115, 116]. Persistent or worsening right upper quadrant abdominal pain and rising liver function studies, including bilirubin and hepatic encephalopathy, are some of the clinical findings related to developing liver ischemia. Computer tomography (CT) and magnetic resonance imaging (MRI) can show the extent of ischemic injury once the diagnosis is suspected.

Liver failure after TIPS placement is thought to be related to the sudden changes in the portosystemic pressure gradient related to shunt placement [114]. Rising bilirubin levels are a sign of worsening liver disease and failure. Avoidance of critically low portosystemic pressure gradients after TIPS, which are associated with fatal complications, is essential in preventing liver failure [116]. Hepatic ischemia or failure related to portosystemic shunting may be treated with shunt caliber reduction.

One of the earliest concerns for stent-graft placement was occlusion of the hepatic veins with their potential occlusion and worsening of liver function. To avoid such complications, initially the stent-graft was deployed only for tract coverage up to the hepatic vein but tract stenosis at the hepatic vein caused shunt dysfunction. Thus, complete coverage from the portal vein to the inferior vena cava was seen as the only possibility to achieve uninterrupted patency. In animal studies, the distribution of the hepatic arterial blood flow is affected by creation of a TIPS with a stent-graft but no relevant concerns were drawn from this experimental study. In the feasibility trials, there was one report describing hepatic necrosis [117, 118]. Overall, only few cases have been described over the last years; unfortunately, it seems that with stent-graft there seems to be a tendency to report more occurrences of liver ischemia or necrosis after TIPS. Episodes of infarcts or necrosis have been reported after initial wedged portograms as well as TIPS creation with bare stents [116–119]. In the TIPS quality improvement trial, the incidence of hepatic infarction was considered to be less than 0.5% [49].

## 3. Personal Experience

From January 2000, we started our experience using the Viatorr stent-graft and following the initial great results the use of this device become a routine in our daily practice. From January 2000 to January 2012, 379 consecutive patients (mean age 52 ± 13 years) underwent de novo TIPS for acute or recurrent variceal bleeding, refractory ascites, hepatic hydrothorax, and Budd-Chiari syndrome.

TIPS placement was due to acute or recurrent variceal bleeding (54.6) and gastric varices (8.0%), refractory ascites (37.9%), hepatic hydrothorax (1.2%), and Budd-Chiari syndrome (6.3%). Cirrhosis was the main cause of portal hypertension (98.9%) and it was related to viral hepatitis, excessive chronic ethanol consumption, cryptogenic hepatitis, and Budd-Chiari syndrome (*N* = 22). According to the Child-Pugh classification, more than half population was class B but also class A (18.4%) and class C (19%). Mean MELD score was 9.5.

All procedures were performed using the Viatorr stent-graft (WL Gore & Associates, Flagstaff, AZ, USA).

Mean follow-up was 37.18 ± 21.94 months long.

Technical success was achieved in all cases (100%). Hemodynamic success (PSG < 12 mm Hg) was achieved in 90.1% of the population. Portosystemic gradient mean value dropped from 21.4 ± 5.4 mm Hg to 7.5 ± 2.9 mm Hg with a statistically significant mean decrease percentage (*P* = 0.0001). In 32 patients treated for refractory ascites, symptoms disappeared but PSG remained slightly high, 13.2 ± 1.35 mm Hg mean value (range 12.1–16 mm Hg), despite the use of a 10 mm diameter stent-graft.

The 30-day mortality rate was 9.2%. Late mortality was 35.4% because of progressive liver failure, multiorgan failure, complacencies due to hepatocellular carcinoma (HCC) onset, hepatorenal syndrome, bleeding, and no pathology-related causes.

After TIPS, 18.9% patients underwent orthotopic liver transplant (OLT) because their clinical conditions had notably improved.

Primary patency rate was 87.93% and secondary patency rate was 97.41%. We observed a considerable reduction in the number of revisions per patient. In fact, the majority of patients required only one revision and only a few (2.07%) needed two revisions. Shunt malfunction was caused by stenosis at the level of the hepatic vein (54.8%), by intraparenchymal stent stenosis (2.3%) and by stenosis at the level of the portal vein (19.0%). An increased PSG value without any real evidence of shunt dysfunction was observed in 23.9% of the cases.

## 4. Conclusions

It can be affirmed that with the passing of time TIPS has been steadily increasing its popularity, and thanks to a significant technological progress it can today offer patients safe and successful outcomes.

Since the time when the first TIPS was performed on a human patient, results have been steadily improving overall thanks to the introduction of covered stents that have almost made restenosis disappear, the main complication of this procedure. As a consequence not only patients' prognosis but also patients' quality of life has become significantly better.

But TIPS remains still today a difficult procedure that can be performed only by expert hands. In fact a learning curve is always mandatory and in particular in case of a blind puncture of the portal vein.

We hope that technical expertise may further improve in the near future and make this procedure easier and consequently more widespread.
